# Cell recruitment in gastric carcinogenesis

**DOI:** 10.18632/aging.101164

**Published:** 2017-01-25

**Authors:** Songhua Zhang, Steven F Moss

**Affiliations:** Division of Gastroenterology, Rhode Island Hospital, Alpert Medical School of Brown University, Providence, RI 02903, USA

**Keywords:** Helicobacter pylori, gastric cancer, bone marrow cells, gastric epithelial cells, p27-deficient mouse

Gastric cancer is the 5^th^ most common malignant disease and the 3^rd^ leading cause of cancer death worldwide. Like most cancers, incidence increases with age. Unlike most other cancers a dominant environmental cause has been established, as chronic infection by *Helicobacter pylori* contributes about 90% of the attributable risk in “non-cardia” gastric cancer, the most common type globally [1].

The development of gastric cancer is a decades-long process, and usually follows an established sequence of progressive histopathological changes from chronic gastritis (occurring soon after *H. pylori* acquisition in childhood) through glandular atrophy, intestinal metaplasia, dysplasia and invasive cancer. This cascade was first described even before *H. pylori* was appreciated as the initial stimulus in this process [2].

The mechanisms underlying the contribution of *H. pylori* to gastric carcinogenesis have been investigated intensely since *H. pylori* was classified by the WHO as a definite carcinogen in 1994. Increased virulence among *H. pylori* strains is conferred by carriage of the cytotoxin-associated gene (cag) pathogenicity island, as the cagA gene encodes an oncogenic CagA protein that can be translocated into gastric epithelial cells to promote tumorigenic activity.

In an important and provocative publication, Houghton *et al* provided evidence in a mouse model that bone marrow-derived cells (BMDC) recruited to the gastric mucosa inflamed by chronic *Helicobacter* infection might become the cells that comprise the resultant gastric dysplasia [3]. Another group has confirmed the incorporation of BMDCs into 25% of the dysplastic gastric glands in mice infected by a murine-adapted human *H. pylori* strain [4]. However, the absence of BMDCs in most of the dysplastic gastric lesions [4] and the failure of progression from dysplasia to cancer in most mouse models, indicates the need to consider additional contributory factors, genetic, environmental or age-related, when testing the gastric BMDC-cancer hypothesis.

We recently explored the contribution of BMDCs to gastric cancer induced by *H. pylori* in p27-deficient mice, since in this model *H. pylori* infection slowly promotes gastric cancer in the mouse equivalent of middle to old age after progressing through the histological intermediaries noted in humans. The goal of our recently published work was to determine whether the loss of p27 expression in bone marrow-derived cells (in a background of wild type epithelium) or loss of p27 expression in gastric epithelial cells (with wild type BM cells) was the cause of the increased gastric cancer susceptibility of these mice [5]. For this purpose, we generated 3 types of chimeric mice through bone marrow transplantation at a young age, established chronic *H. pylori* infection by gavage, and evaluated outcomes 1 year later, at an average age of 62-64 weeks. Autologous wild type to wild type trans-plantation served as a control for the 2 experimental groups: p27 knock-out bone marrow transplanted into wild type recipients and wild type bone marrow transplanted into p27-deficient recipients. (Table). The donor origins of the cells in the gastric mucosa were tracked through a combination of Y chromosome analysis and/or green fluorescent protein expression, with E-cadherin staining to demonstrate epithelial differentiation. We found that although the autologous control mice exhibited chronic gastric inflammation this did not progress to neoplasia. In contrast, mice in both of the other 2 arms of the study exhibited severe gastritis and some in each group developed gastric epithelial dysplasia (not significantly different between the p27-deficient mice that received wild type marrow versus the reverse transplant). BMDCs with epithelial phenotypes were observed in the gastric mucosa of mice in all groups, including, and at high frequency, in the dysplastic lesions of the two heterologous groups demonstrating that bone marrow-derived and gastric epithelial cells contributed to the gastric cancer development in these *H. pylori*-infected p27 deficient mice. There were distinct proinflam-matory cytokine/chemokine profiles in each of the 2 experimental groups, indicating that although bone marrow-derived and gastric epithelial cells both had a role in the increased gastric cancer susceptibility, oncogenesis may be mediated via two distinct pathways operative in *H. pylori*-infected p27-deficient mice [5]. Further investigation of the potential dysregulation of gastric progenitor and stem cells in aged mice and exploration of their role in *H. pylori*-related cancer are awaited.

**Table:** Predicted gastric cancer outcomes following bone marrow transplant and *H. pylori* infection in mice

Donor	Recipient	Expected Gastric Cancer Incidence if Cancer Due to p27 Deficiency in Bone Marrow Cells	Expected Gastric Cancer Incidednce if Cancer Due to p27 Deficiency in Gastric Epithelial Cells
GFP+ Wild Type	Wild Type	Low	Low
p27 −/−	Wild Type	High	Low
GFP+ Wild Type	p27 −/−	Low	High

While studies of this type may be of interest mechanistically for inflammation-associated cancers in general, investigating the inflammatory origins of gastric cancer may also have direct clinical utility in the era of cancer immunotherapy. Current *H. pylori* eradication therapy reduces the risk of gastric cancer [6], but increasing antibiotic resistance is limiting our ability to achieve complete *H. pylori* elimination, especially in high prevalence areas. Vaccination against *H. pylori* at a young age may be a cost-effective alternative strategy to prevent *H. pylori*-related diseases in adulthood, as suggested by the results of a large clinical trial recently completed in China [7]. Given the continued poor outcomes of patients diagnosed with gastric cancer, an emphasis on early detection and effective prevention should help decrease the mortality rate of gastric cancer in an aging world population.
